# M2PP: a novel computational model for predicting drug-targeted pathogenic proteins

**DOI:** 10.1186/s12859-021-04522-9

**Published:** 2022-01-04

**Authors:** Shiming Wang, Jie Li, Yadong Wang

**Affiliations:** School of Computer Science and Technology, Harbin Institute of Technology, Harbin, Heilongjiang 150001 China

**Keywords:** Disease, Pathogenic proteins, Target, Prediction

## Abstract

**Background:**

Detecting pathogenic proteins is the origin way to understand the mechanism and resist the invasion of diseases, making pathogenic protein prediction develop into an urgent problem to be solved. Prediction for genome-wide proteins may be not necessarily conducive to rapidly cure diseases as developing new drugs specifically for the predicted pathogenic protein always need major expenditures on time and cost. In order to facilitate disease treatment, computational method to predict pathogenic proteins which are targeted by existing drugs should be exploited.

**Results:**

In this study, we proposed a novel computational model to predict drug-targeted pathogenic proteins, named as M2PP. Three types of features were presented on our constructed heterogeneous network (including target proteins, diseases and drugs), which were based on the neighborhood similarity information, drug-inferred information and path information. Then, a random forest regression model was trained to score unconfirmed target-disease pairs. Five-fold cross-validation experiment was implemented to evaluate model’s prediction performance, where M2PP achieved advantageous results compared with other state-of-the-art methods. In addition, M2PP accurately predicted high ranked pathogenic proteins for common diseases with public biomedical literature as supporting evidence, indicating its excellent ability.

**Conclusions:**

M2PP is an effective and accurate model to predict drug-targeted pathogenic proteins, which could provide convenience for the future biological researches.

## Background

Overcoming diseases is the eternal goal of human beings, and the current treatment strategies mainly depend on drugs, aiming to act on the target genes or proteins to alleviate the symptoms or even prevent the attack of the disease [1]. In the drug-target-disease mechanism, identifying the disease-caused protein is a crucial and fundamental problem, also becomes challenge at the same time [2]. Currently, computational methods to predict pathogenic targets have been widely applied because of their high efficiency and low consumption prior to in vitro or in vivo biological experimental methods [3]. During the past decades, various prediction methods have been presented with different performances.

Earlier researches mainly focused on the protein–protein interaction (PPI) network, whose topological structure was directly used to predict disease-gene associations [4, 5]. However, the large number of false positives in the PPI network from public databases made these methods difficult to acquire higher prediction accuracy. Hence, the disease-related clinical data was added into later studies, which were based on GWAS [6–8] and gene expression [9–13], respectively. Although these methods obtained more accurate prediction than methods which applied PPI network alone, limitations still existed. For example, even the comprehensive platform TCGA [14] could only provide limited available data about uncommon cancers, let alone other non-cancer diseases, which greatly restricted the performance of these methods. Difficult to break limitations on the data source, researchers have begun to conduct in-depth research on algorithms, where the most widely used were about machine learning. Model GCN-MF combined the graph convolutional network with matrix factorization for disease-gene association identification [15]. Natarajan et al. derived features of diseases and genes for the inductive matrix completion [16]. Method CATAPULT was proposed by training a biased support vector machine model with features derived from a heterogeneous network [17]. Zeng et al. considered this problem as the recommender system, presenting a probability-based collaborative filtering model to predict pathogenic human genes [18]. Luo et al. developed a method to predict disease–gene associations with multimodal deep learning [19]. Although these efforts on algorithm development made prediction results improved, most methods still extracted valid information only from gene data and disease data. Actually, utilizing other information besides gene and disease to solve the prediction problem is essential and urgent in such intricate biological networks.

The ultimate objective of predicting pathogenic genes or proteins is to find a breakthrough for disease treatment. If predicting on the whole gene (protein) set, even though a novel gene-disease (protein-disease) association is successfully predicted, it will still be a long process to treat the disease specifically for this gene (protein). The reason comes from many aspects, for example, the research and development for new drugs usually take a long time. Actually, reducing the scope of the whole protein set to drug-targeted protein set will be more conducive for the disease treatment in clinical research, because for a novel predicted protein-disease association, the drugs which target this protein can be regarded as a candidate collection for the disease treatment instead of developing new drugs. Hence, we proposed a method to predict drug-targeted pathogenic proteins, named as M2PP. First, the target, disease and drug set were collected to construct association networks and similarity networks. Then, features were constructed for each target-disease pair based on the neighborhood similarity information, drug-inferred information and path information, respectively. Finally, a random forest regression model was trained to score unconfirmed target-disease pairs.

## Method

### Data collection

**Fig. 1:**
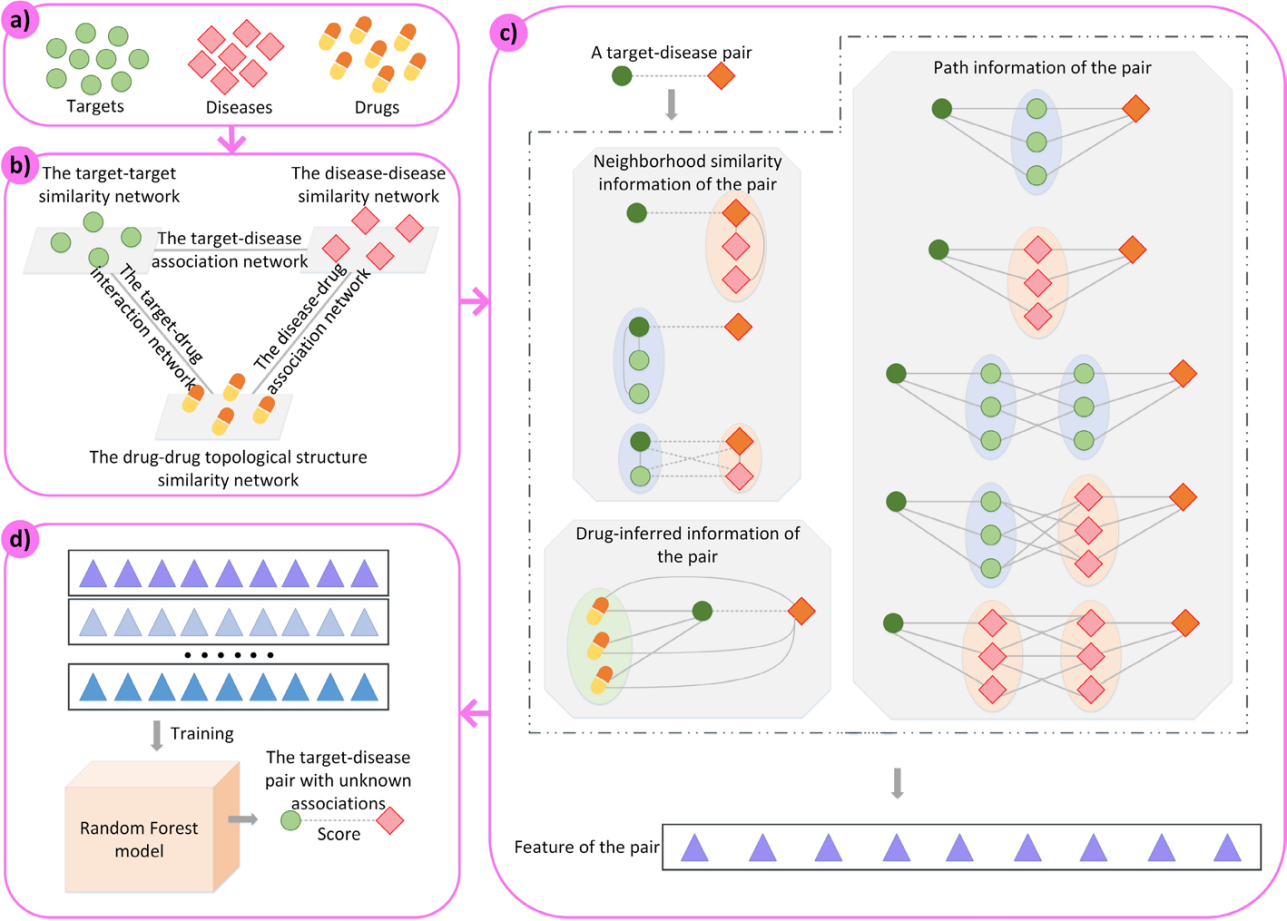
The framework of M2PP. **a** Construct the target set, disease set and drug set; **b** Construct heterogeneous networks: the target-disease association network, target-drug interaction network, disease-drug association network, disease-disease similarity network, target-target similarity network and drug-drug topological structure similarity network; **c** Construct features for target-disease pairs; **d** Train the random forest model and predict association scores for unconfirmed target-disease pairs

We collected the drug-targeted single human target proteins from DrugBank [20], where the drugs were approved by the Food and Drug Administration (FDA) [21]. For these targets, we extracted diseases which had curated associations with them from the Comparative Toxicogenomics Database (CTD) [22]. Then, three sets (a target set, a disease set and a drug set) were constructed. Next, we reduced these sets to make sure that any element in one set had association with both the other two sets (all associations were from DrugBank and CTD). Finally, we obtained 1002 targets, 1035 diseases and 1095 drugs (Fig. 1a)). The target set, disease set and drug set were represented as $$T = \left\{ {t_{1} ,t_{2} , \ldots ,t_{nT} } \right\}.$$$${\text{ D}} = \left\{ {{\text{d}}_{1} ,{\text{d}}_{2} , \ldots ,{\text{d}}_{{{\text{nD}}}} } \right\}$$ and $${\text{ M}} = \left\{ {{\text{m}}_{1} ,{\text{m}}_{2} , \ldots ,{\text{m}}_{{{\text{nM}}}} } \right\}$$, respectively.

### Network construction

First, we constructed three association networks among the target, disease and drug set: (1) the target-disease association network, including 7342 curated associations from CTD, whose adjacency matrix was represented as ${\text{TDA}}^{{{\text{nT}} \times {\text{nD}}}}$; (2) the target-drug interaction network, including 38,871 curated interactions from DrugBank and CTD, representing its adjacency matrix as ${\text{TDI}}^{{{\text{nT}} \times {\text{nM}}}}$; (3) the disease-drug association network, including 35,319 curated associations from CTD, with adjacency matrix of ${\text{DDA}}^{{{\text{nD}} \times {\text{nM}}}}$. For target ${\text{ t}}_{{\text{i}}}$ $\left( {1 \le {\text{i}} \le {\text{nT}}} \right)$ and disease ${\text{d}}_{{\text{j}}}$ $\left( {1 \le {\text{j}} \le {\text{nD}}} \right)$, if the known association between them was existed,${\text{ TDA}}_{{{\text{i}},{\text{j}}}} = 1$; otherwise,${\text{ TDA}}_{{{\text{i}},{\text{j}}}} = 0$. Analogously did ${\text{ TDI }}$ and ${\text{ DDA}}$.

Then, we constructed the similarity networks:

(1) The disease-disease similarity network. We calculated the disease semantic similarities based on the Medical Subject Headings (MESH) descriptors [23] by the IDSSIM algorithm [24] and based on Disease Ontology (DO) [25] by Wang et al.’s method [26], respectively. For a disease-disease pair, the mean value of the two similarities was computed to construct the semantic similarity matrix ${\text{DDS}}\_{\text{S}}^{{{\text{nD}} \times {\text{nD}}}}$. Then, we calculated diseases’ topological structure similarity [27], whose matrix was represented as ${\text{DDS}}\_{\text{T}}^{{{\text{nD}} \times {\text{nD}}}}$:

1 $${\text{DDS}}\_{\text{T}}_{i,j} = {\text{exp}}\left( { - \alpha ||TDA_{,i} - TDA_{,j}||^{2} } \right)$$

$$\alpha = \alpha ^{\prime } /\frac{1}{{nD}}\sum\limits_{{k = 1}}^{{nD}} {||TDA_{,k} ||} ^{2}$$

where $1 \le {\text{i}},{\text{j}} \le {\text{nD}}$; $TDA_{,i}$ was the *i*th column of $TDA$; $\alpha^{\prime}$ was set to 1 according to previous study [28]. For the two similarity matrices ${\text{DDS}}\_{\text{S}}$ and ${\text{DDS}}\_{\text{T}}$, we proposed an integration way based on the entropy to get the final disease similarity matrix ${\text{DDS}}^{{{\text{nD}} \times {\text{nD}}}}$. The entropy of row $i$ in matrix ${\text{W}}^{x \times y}$ was represented as ${\text{E}}_{i}^{{\text{W }}}$:

2 $${\text{E}}_{i}^{{\text{W }}} = - \mathop \sum \limits_{j = 1}^{y} p_{i,j} {\text{log}}\left( {p_{i,j} } \right)$$

$$p_{{i,j}} = {{{\text{W}}_{{i,j}} } \mathord{\left/ {\vphantom {{{\text{W}}_{{i,j}} } {\sum\limits_{{k = 1}}^{y} {{\text{W}}_{{i,k}} } }}} \right. \kern-\nulldelimiterspace} {\sum\limits_{{k = 1}}^{y} {{\text{W}}_{{i,k}} } }}$$

According to the formula above, the entropy of disease ${\text{d}}_{{\text{i}}}$ in matrix ${\text{DDS}}\_{\text{S}}$ and ${\text{DDS}}\_{\text{T}}$ was calculated and represented as ${\text{E}}_{i}^{{{\text{DDS}}\_{\text{S }}}}$ and ${\text{E}}_{i}^{{{\text{DDS}}\_{\text{T }}}}$, respectively. All diseases could be divided into two subsets, ${\text{D}}\_{\text{A}}$ and ${\text{D}}\_{\text{B}}$:

3 $${\text{D}}\_{\text{A}} = \left\{ {{\text{d}}_{{\text{i}}} {\text{|E}}_{i}^{{{\text{DDS}}\_{\text{S }}}} \le {\text{E}}_{i}^{{{\text{DDS}}\_{\text{T }}}} ,1 \le {\text{i}} \le {\text{nD}}} \right\}$$

4 $${\text{D}}\_{\text{B}} = \left\{ {{\text{d}}_{{\text{j}}} {\text{|E}}_{j}^{{{\text{DDS}}\_{\text{T }}}} < {\text{E}}_{j}^{{{\text{DDS}}\_{\text{S }}}} ,1 \le {\text{j}} \le {\text{nD}}} \right\}$$

The similarity matrix ${\text{DDS}}$ could be divided into four parts by ${\text{D}}\_{\text{A}}$ and ${\text{D}}\_{\text{B}}$:

5 $${\text{DDS}} = \left[ {\begin{array}{*{20}c} {{\text{similarity matrix between D}}\_{\text{A and }} {\text{D}}\_{\text{A}}} & {{\text{similarity matrix between D}}\_{\text{A and }} {\text{D}}\_{\text{B}}} \\ {{\text{similarity matrix between D}}\_{\text{B and }} {\text{D}}\_{\text{A}}} & {{\text{similarity matrix between D}}\_{\text{B and }} {\text{D}}\_{\text{B}}} \\ \end{array} } \right]$$

A low entropy value meant little random information from the similarities. Hence, the upper left and lower right part of ${\text{DDS}}$ were defined as below:

6 $${\text{similarity matrix between D}}\_{\text{A and }} {\text{D}}\_{\text{A}} = {\text{DDS}}\_{\text{S}}_{{{\text{D}}\_{\text{A}},{\text{D}}\_{\text{A}}}}$$

7 $${\text{similarity matrix between D}}\_{\text{B and }} {\text{D}}\_{\text{B}} = {\text{DDS}}\_{\text{T}}_{{{\text{D}}\_{\text{B}},{\text{D}}\_{\text{B}}}}$$

The similarities between ${\text{D}}\_{\text{A}}$ and ${\text{D}}\_{\text{B}}$ were still integrated based on the entropy. ${\text{D}}\_{\text{A}}$ was divided into two subsets, ${\text{D}}\_{\text{A}}\_{\text{a}}$ and ${\text{D}}\_{\text{A}}\_{\text{b}}$:

8 $${\text{D}}\_{\text{A}}\_{\text{a}} = \left\{ {{\text{d}}_{{\text{i}}} {\text{|E}}_{i}^{{{\text{DDS}}\_{\text{S}}_{{{\text{D}}\_{\text{A}},{\text{D}}\_{\text{B}}}} { }}} \le {\text{E}}_{i}^{{{\text{DDS}}\_{\text{T}}_{{{\text{D}}\_{\text{A}},{\text{D}}\_{\text{B}}}} { }}} ,1 \le {\text{i}} \le \left| {{\text{D}}\_{\text{A}}} \right|} \right\}$$

9 $${\text{D}}\_{\text{A}}\_{\text{b}} = \left\{ {{\text{d}}_{{\text{j}}} {\text{|E}}_{j}^{{{\text{DDS}}\_{\text{T}}_{{{\text{D}}\_{\text{A}},{\text{D}}\_{\text{B}}}} { }}} < {\text{E}}_{j}^{{{\text{DDS}}\_{\text{S}}_{{{\text{D}}\_{\text{A}},{\text{D}}\_{\text{B}}}} { }}} ,1 \le {\text{j}} \le \left| {{\text{D}}\_{\text{A}}} \right|} \right\}$$

The ${\text{similarity matrix between D}}\_{\text{A and }} {\text{D}}\_{\text{B}}$ could be represented as below:

10 $${\text{Similarity}}\;{\text{matrix}}\;{\text{between}}\;{\text{D}}\_{\text{A}}\;{\text{and}}\;{\text{D}}\_{\text{B}} = \left[ {\begin{array}{*{20}c} {{\text{DDS}}\_{\text{S}}_{{{\text{D}}\_{\text{A}}\_{\text{a}},{\text{D}}\_{\text{B}}}} } \\ {{\text{DDS}}\_{\text{T}}_{{{\text{D}}\_{\text{A}}\_{\text{b}},{\text{D}}\_{\text{B}}}} } \\ \end{array} } \right]$$

To ensure the symmetry of ${\text{DDS}}$, the ${\text{similarity matrix between D}}\_{\text{B and }} {\text{D}}\_{\text{A}}$ was set as the transpose of ${\text{similarity}}\;{\text{matrix}}\;{\text{between}}\;{\text{D}}\_{\text{A}}\;{\text{and}}\;{\text{D}}\_{\text{B}}$. Finally, ${\text{DDS}}$ could be obtained as below:

11 $${\text{DDS}} = \left[ {\begin{array}{*{20}c} {{\text{DDS}}\_{\text{S}}_{{{\text{D}}\_{\text{A}},{\text{D}}\_{\text{A}}}} } & {\left[ {\begin{array}{*{20}c} {{\text{DDS}}\_{\text{S}}_{{{\text{D}}\_{\text{A}}\_{\text{a}},{\text{D}}\_{\text{B}}}} } \\ {{\text{DDS}}\_{\text{T}}_{{{\text{D}}\_{\text{A}}\_{\text{b}},{\text{D}}\_{\text{B}}}} } \\ \end{array} } \right]} \\ {\left[ {\begin{array}{*{20}c} {{\text{DDS}}\_{\text{S}}_{{{\text{D}}\_{\text{A}}\_{\text{a}},{\text{D}}\_{\text{B}}}} } \\ {{\text{DDS}}\_{\text{T}}_{{{\text{D}}\_{\text{A}}\_{\text{b}},{\text{D}}\_{\text{B}}}} } \\ \end{array} } \right]^{T} } & {{\text{DDS}}\_{\text{T}}_{{{\text{D}}\_{\text{B}},{\text{D}}\_{\text{B}}}} } \\ \end{array} } \right]$$

(2) The target-target similarity network. We calculated the target proteins’ amino acid sequences similarity from the KEGG database [29] by the Smith-Waterman algorithm [30] and the protein functional similarity by Chen et al.’s method [31], respectively. For a target-target pair, the mean value of the two similarities was calculated to construct the similarity matrix ${\text{TTS}}\_{\text{S}}^{{{\text{nT}} \times {\text{nT}}}}$. Then, targets’ topological structure similarity matrix ${\text{TTS}}\_{\text{T}}^{{{\text{nT}} \times {\text{nT}}}}$ was computed as below:

12 $${\text{TTS}}\_{\text{T}}_{i,j} = {\text{exp}}\left( { - \beta ||TDA_{i,} - TDA_{j,}||^{2} } \right)$$

$$\beta = {{\beta ^{\prime } } \mathord{\left/ {\vphantom {{\beta ^{\prime } } {\frac{1}{{nT}}\sum\limits_{{k = 1}}^{{nT}} | |TDA_{{k,}} ||^{2} }}} \right. \kern-\nulldelimiterspace} {\frac{1}{{nT}}\sum\limits_{{k = 1}}^{{nT}} | |TDA_{{k,}} ||^{2} }}$$

where $1 \le {\text{i}},{\text{j}} \le {\text{nT}}$; $TDA_{i,}$ was the *i*th row of $TDA$; $\beta^{\prime} = 1$.

The target subset ${\text{T}}\_{\text{A}}$, ${\text{T}}\_{\text{B}}$, ${\text{T}}\_{\text{A}}\_{\text{a}}$ and ${\text{T}}\_{\text{A}}\_{\text{b}}$ were defined as below:

13 $${\text{T}}\_{\text{A}} = \left\{ {{\text{t}}_{{\text{i}}} {\text{|E}}_{i}^{{{\text{TTS}}\_{\text{S }}}} \le {\text{E}}_{i}^{{{\text{TTS}}\_{\text{T }}}} ,1 \le {\text{i}} \le {\text{nT}}} \right\}$$

14 $${\text{T}}\_{\text{B}} = \left\{ {{\text{t}}_{{\text{j}}} {\text{|E}}_{j}^{{{\text{TTS}}\_{\text{T }}}} < {\text{E}}_{j}^{{{\text{TTS}}\_{\text{S }}}} ,1 \le {\text{j}} \le {\text{nT}}} \right\}$$

15 $${\text{T}}\_{\text{A}}\_{\text{a}} = \left\{ {{\text{t}}_{{\text{i}}} {\text{|E}}_{i}^{{{\text{TTS}}\_{\text{S}}_{{{\text{T}}\_{\text{A}},{\text{T}}\_{\text{B}}}} { }}} \le {\text{E}}_{i}^{{{\text{TTS}}\_{\text{T}}_{{{\text{T}}\_{\text{A}},{\text{T}}\_{\text{B}}}} { }}} ,1 \le {\text{i}} \le \left| {{\text{T}}\_{\text{A}}} \right|} \right\}$$

16 $${\text{T}}\_{\text{A}}\_{\text{b}} = \left\{ {{\text{t}}_{{\text{j}}} {\text{|E}}_{j}^{{{\text{TTS}}\_{\text{T}}_{{{\text{T}}\_{\text{A}},{\text{T}}\_{\text{B}}}} { }}} < {\text{E}}_{j}^{{{\text{TTS}}\_{\text{S}}_{{{\text{T}}\_{\text{A}},{\text{T}}\_{\text{B}}}} { }}} ,1 \le {\text{j}} \le \left| {{\text{T}}\_{\text{A}}} \right|} \right\}$$

Finally, ${\text{TTS}}\_{\text{S}}$ and ${\text{TTS}}\_{\text{T}}$ were integrated into the final target similarity matrix ${\text{TTS}}^{nT \times nT}$:

17 $${\text{TTS}} = \left[ {\begin{array}{*{20}c} {{\text{TTS}}\_{\text{S}}_{{{\text{T}}\_{\text{A}},{\text{T}}\_{\text{A}}}} } & {\left[ {\begin{array}{*{20}c} {{\text{TTS}}\_{\text{S}}_{{{\text{T}}\_{\text{A}}\_{\text{a}},{\text{T}}\_{\text{B}}}} } \\ {{\text{TTS}}\_{\text{T}}_{{{\text{T}}\_{\text{A}}\_{\text{b}},{\text{T}}\_{\text{B}}}} } \\ \end{array} } \right]} \\ {\left[ {\begin{array}{*{20}c} {{\text{TTS}}\_{\text{S}}_{{{\text{T}}\_{\text{A}}\_{\text{a}},{\text{T}}\_{\text{B}}}} } \\ {{\text{TTS}}\_{\text{T}}_{{{\text{T}}\_{\text{A}}\_{\text{b}},{\text{T}}\_{\text{B}}}} } \\ \end{array} } \right]^{T} } & {{\text{TTS}}\_{\text{T}}_{{{\text{T}}\_{\text{B}},{\text{T}}\_{\text{B}}}} } \\ \end{array} } \right]$$

(3) The drug-drug topological structure similarity networks. We calculated drugs’ topological structure similarities in the target-drug interaction network and the disease-drug association network, respectively. They were represented as ${\text{MMS}}\_{\text{T}}^{{{\text{nM}} \times {\text{nM}}}}$ and ${\text{ MMS}}\_{\text{D}}^{{{\text{nM}} \times {\text{nM}}}}$, respectively:

18 $${\text{MMS}}\_{\text{T}}_{{i,j}} = {\text{exp}}\left( { - \gamma ||TDI_{{,i}} - TDI_{{,j}} ||^{2} } \right)$$

$$\gamma = {{\gamma ^{\prime } } \mathord{\left/ {\vphantom {{\gamma ^{\prime } } {\frac{1}{{nM}}\sum\limits_{{k = 1}}^{{nM}} {||TDI_{{,k}} ||^{2} } }}} \right. \kern-\nulldelimiterspace} {\frac{1}{{nM}}\sum\limits_{{k = 1}}^{{nM}} {||TDI_{{,k}} ||^{2} } }}$$

19 $${\text{MMS}}\_{\text{D}}_{{i,j}} = {\text{exp}}\left( { - \delta ||DDA_{{,i}} - DDA_{{,j}} ||^{2} } \right)$$

$$\delta = {{\delta ^{\prime } } \mathord{\left/ {\vphantom {{\delta ^{\prime } } {\frac{1}{{nM}}\sum\limits_{{k = 1}}^{{nM}} {||DDA_{{,k}} ||^{2} } }}} \right. \kern-\nulldelimiterspace} {\frac{1}{{nM}}\sum\limits_{{k = 1}}^{{nM}} {||DDA_{{,k}} ||^{2} } }}$$

where $1 \le {\text{i}},{\text{j}} \le {\text{nM}}$; $TDI_{,i}$ and $DDA_{,i}$ was the *i*th column of $TDI$ and $DDA$, respectively; $\gamma^{\prime} = 1$;$\delta^{\prime} = 1$.

Finally, the heterogeneous network was constructed as shown in (Fig. 1b)). The characteristics of data in these networks were summarized in Table 1, where the sparsity was the ratio of edges to the network size. Obviously, our objective network (the target-disease association network) was the most imbalanced.

**Table 1:** The instruction of the five networks’ characteristics

Network	Size of the network	Number of the edges	Range of the edges’ weight	Sparsity
The target-disease association network	1002*1035	7342	0 or 1	0.007
The target-drug interaction network	1002*1095	38,871	0 or 1	0.035
The disease-drug association network	1035*1095	35,319	0 or 1	0.031
The disease-disease similarity network	1035*1035	1,071,225	[0,1]	1
The target-target similarity network	1002*1002	1,004,004	[0,1]	1
The drug-drug topological structure similarity network	1095*1095	1,199,025	[0,1]	1

### Feature construction for model training to score unconfirmed target-disease pairs

For target-disease pair ${\text{ t}}_{{\text{i}}}$-${\text{ d}}_{{\text{j}}}$ ($1 \le {\text{i}} \le {\text{nT}},{ }1 \le {\text{j}} \le {\text{nD}}$), we constructed a 9-dimension feature based on its neighborhood similarity information, drug-inferred information and path information (Fig. 1c)), shown in the following formulas:

20 $${\text{Fea}}1 = {\text{mean}}\left( {{\text{DDS}}_{{{\text{P}},{\text{j}}}} } \right)$$

$${\text{P}} = \left\{ {{\text{y| TDA}}_{{{\text{i}},{\text{y}}}} = 0,{ }1 \le {\text{y}} \le {\text{nD}}} \right\}$$

21 $${\text{Fea}}2 = {\text{mean}}\left( {{\text{TTS}}_{{{\text{i}},{\text{Q}}}} } \right)$$

$${\text{Q}} = \left\{ {{\text{x| TDA}}_{{{\text{x}},{\text{j}}}} = 0,{ }1 \le {\text{x}} \le {\text{nT}}} \right\}$$

22 $${\text{Fea}}3 = {\text{TTS}}_{{{\text{i}},{\text{a}}}} \times {\text{TDA}}_{{{\text{a}},{\text{j}}}} + {\text{TDA}}_{{{\text{i}},{\text{b}}}} \times {\text{DDS}}_{{{\text{b}},{\text{j}}}} + {\text{TTS}}_{{{\text{i}},{\text{a}}}} \times {\text{TDA}}_{{{\text{a}},{\text{b}}}} \times {\text{DDS}}_{{{\text{b}},{\text{j}}}}$$

$${\text{ a}} = \mathop {\text{arg max}}\limits_{{\text{x}}} {\text{TTS}}_{{{\text{i}},{\text{x}} = \left\{ {1,2, \ldots {\text{nT}}} \right\}\backslash {\text{i}}}}$$

$${\text{ b}} = \mathop {\text{arg max}}\limits_{{\text{y}}} {\text{DDS}}_{{{\text{y}} = \left\{ {1,2, \ldots {\text{nD}}} \right\}\backslash {\text{j}},{\text{j}}}}$$

23 $${\text{Fea}}4 = \mathop {\max }\limits_{{{\text{k}} \in {\text{K}}}} \left( {{\text{L}}_{{{\text{j}},{\text{k}}}} /{\text{H}}_{{{\text{i}},{\text{k}}}} } \right)$$

$${\text{K}} = \left\{ {{\text{z| TDI}}_{{{\text{i}},{\text{z}}}} = 1,{\text{DDA}}_{{{\text{j}},{\text{z}}}} = 1,{ }1 \le {\text{z}} \le {\text{nM}}} \right\}$$

$${\text{ H}}_{{{\text{i}},{\text{k}}}} = \left( {{\text{TDI}} \times {\text{MMS}}\_{\text{T}}} \right)_{{{\text{i}},{\text{k}}}} /\left| {\left\{ {{\text{x|TDI}}_{{{\text{i}},{\text{x}}}} = 1,{\text{ MMS}}\_{\text{T}}_{{{\text{x}},{\text{k}}}} \ne 0,{ }1 \le {\text{x}} \le {\text{nM}}} \right\}} \right|$$

$${\text{ L}}_{{{\text{j}},{\text{k}}}} = \left( {{\text{DDA}} \times {\text{MMS}}\_{\text{D}}} \right)_{{{\text{j}},{\text{k}}}} /\left| {\left\{ {{\text{y|DDA}}_{{{\text{j}},{\text{y}}}} = 1,{\text{ MMS}}\_{\text{D}}_{{{\text{y}},{\text{k}}}} \ne 0,{ }1 \le {\text{y}} \le {\text{nM}}} \right\}} \right|$$

24 $${\text{Fea}}5 = \left( {{\text{TTS}} \times {\text{TDA}}} \right)_{{{\text{i}},{\text{j}}}} /\left| {\left\{ {{\text{x|TTS}}_{{{\text{i}},{\text{x}}}} \ne 0,{\text{ TDA}}_{{{\text{x}},{\text{j}}}} = 1,{ }1 \le {\text{x}} \le {\text{nT}}} \right\}} \right|$$

25 $${\text{Fea}}6 = \left( {{\text{TDA}} \times {\text{DDS}}} \right)_{{{\text{i}},{\text{j}}}} /\left| {\left\{ {{\text{y|TDA}}_{{{\text{i}},{\text{y}}}} = 1,{\text{ DDS}}_{{{\text{y}},{\text{j}}}} \ne 0,{ }1 \le {\text{y}} \le {\text{nD}}} \right\}} \right|$$

26 $${\text{Fea}}7 = \frac{{\left( {{\text{TTS}} \times {\text{TTS}} \times {\text{TDA}}} \right)_{{{\text{i}},{\text{j}}}} }}{{\left| {\left\{ {\left( {{\text{x}},{\text{s}}} \right){\text{|TTS}}_{{{\text{i}},{\text{x}}}} \ne 0,{\text{ TTS}}_{{{\text{x}},{\text{s}}}} \ne 0,{\text{ TDA}}_{{{\text{s}},{\text{j}}}} = 1,{ }1 \le {\text{x}},{\text{s}} \le {\text{nT}}} \right\}} \right|}}$$

27 $${\text{Fea}}8 = \frac{{\left( {{\text{TTS}} \times {\text{TDA}} \times {\text{DDS}}} \right)_{{{\text{i}},{\text{j}}}} }}{{\left| {\left\{ {\left( {{\text{x}},{\text{y}}} \right){\text{|TTS}}_{{{\text{i}},{\text{x}}}} \ne 0,{\text{ TDA}}_{{{\text{x}},{\text{y}}}} = 1,{\text{ DDS}}_{{{\text{y}},{\text{j}}}} \ne 0,{ }1 \le {\text{x}} \le {\text{nT}},1 \le {\text{y}} \le {\text{nD}}} \right\}} \right|}}$$

28 $${\text{Fea}}9 = \frac{{\left( {{\text{TDA}} \times {\text{DDS}} \times {\text{DDS}}} \right)_{{{\text{i}},{\text{j}}}} }}{{\left| {\left\{ {\left( {{\text{y}},{\text{t}}} \right){\text{|TDA}}_{{{\text{i}},{\text{y}}}} = 1,{\text{ DDS}}_{{{\text{y}},{\text{t}}}} \ne 0,{\text{DDS}}_{{{\text{t}},{\text{j}}}} \ne 0,{ }1 \le {\text{y}},{\text{t}} \le {\text{nD}}} \right\}} \right|}}$$

The analysis of these features were summarized in Table 2, including each feature’s type, description, content and information source. Considering each target-disease pair in the training set as a sample, the pair with known associations was regarded as a positive sample which was labelled as 1, while the pair which did not have known associations was regarded as a negative sample labelled as 0. After constructing features for each sample, the training set was used to train the random forest regression model [32], then the prediction model was used to score the unconfirmed target-disease pairs (Fig. 1d)). A higher score represented a larger possibility that the unconfirmed pair was associated. Parameters of mtry and ntree in the random forest model were set to 3 (the number of features/3) and 500 according to the default settings in R package, respectively.

**Table 2:** Information summary of the constructed features and their influence coefficient

Type	Description	Feature	Content	Information source	Influence coefficient
The neighborhood similarity information	Information based on the similarities between the specific disease (target) and its neighborhoods	$\mathrm{Fea}1$	The average similarity between the specific disease and its neighborhoods which did not have known associations with the specific target	$\text{DDS TDA}$	0.58
		$\mathrm{Fea}2$	The average similarity between the specific target and its neighborhoods which did not have known associations with the specific disease	$\text{TTS TDA}$	0.576
		$$\mathrm{Fea}3$$	The sum of weights for paths which connected by the nearest neighborhood of the specific target and the nearest neighborhood of the specific disease	$$\text{TTS DDS }$$$$\mathrm{TDA}$$	0.638
The drug-inferred information	Information inferred by drugs based on the drug-target-disease mechanism	$\mathrm{Fea}4$	The maximum quotient of the average weight for the specific disease-drug paths divided by the average weight for the specific target-drug paths	$\text{TDI DDA }{\mathrm{MMS}}\_{\mathrm{T}}\text{ MMS}\_\mathrm{D}$	0.671
The path information	Information from paths (length = 2 and length = 3) between the specific target and the specific disease	$\mathrm{Fea}5$	The average weight of paths from the specific target to the specific disease based on target-target-disease pattern	$\text{TTS TDA}$	0.745
		$\mathrm{Fea}6$	The same as above but based on target-disease-disease pattern	${\text{DDS TDA}}$	0.722
		${\text{Fea}}7$	The same as above but based on target-target-target-disease pattern	${\text{TTS TDA}}$	0.671
		${\text{Fea}}8$	The same as above but based on target-target-disease-disease pattern	${\text{TTS DDS TDA}}$	0.654
		${\text{Fea}}9$	The same as above but based on target-disease-disease-disease pattern	${\text{DDS TDA}}$	0.593

## Results

### Evaluation metric

The fivefold cross-validation (CV) experiment was implemented to evaluate the performance of diverse prediction models. In the target-disease association network, there were 7342 known associations and 1,029,728 unconfirmed pairs. First, the 7342 target-disease associations and 7342 randomly selected unconfirmed pairs were considered as positive samples and negative samples, respectively. The remaining 1,022,386 unconfirmed pairs was unlabeled samples. Then, the positive samples and negative samples were evenly divided into 5 parts, where each part contained the same amount of positive and negative samples. In each CV, four parts were taken as training set in turn to train the model, while the remaining part and all unlabeled samples were taken as test set. For each test sample, the model could give a score representing the possibility that the pair was associated. We calculated the true positive rate (TPR) and false positive rate (FPR) for these scores under different thresholds to acquire the areas under the receiver operating characteristic curve (AUROC) and the areas under the precision–recall curve (AUPR). In fivefold CV, we obtained five AUROC/AUPR values and adopted the average AUROC/AUPR value to evaluate the performance of the model in this CV. To make the results more reliable, we repeated fivefold CV for 5 times to compute the mean and standard deviation (SD) values of the five average AUROC/AUPR values as the final evaluation metrics for prediction models.

### Feature analysis

**Fig. 2:**
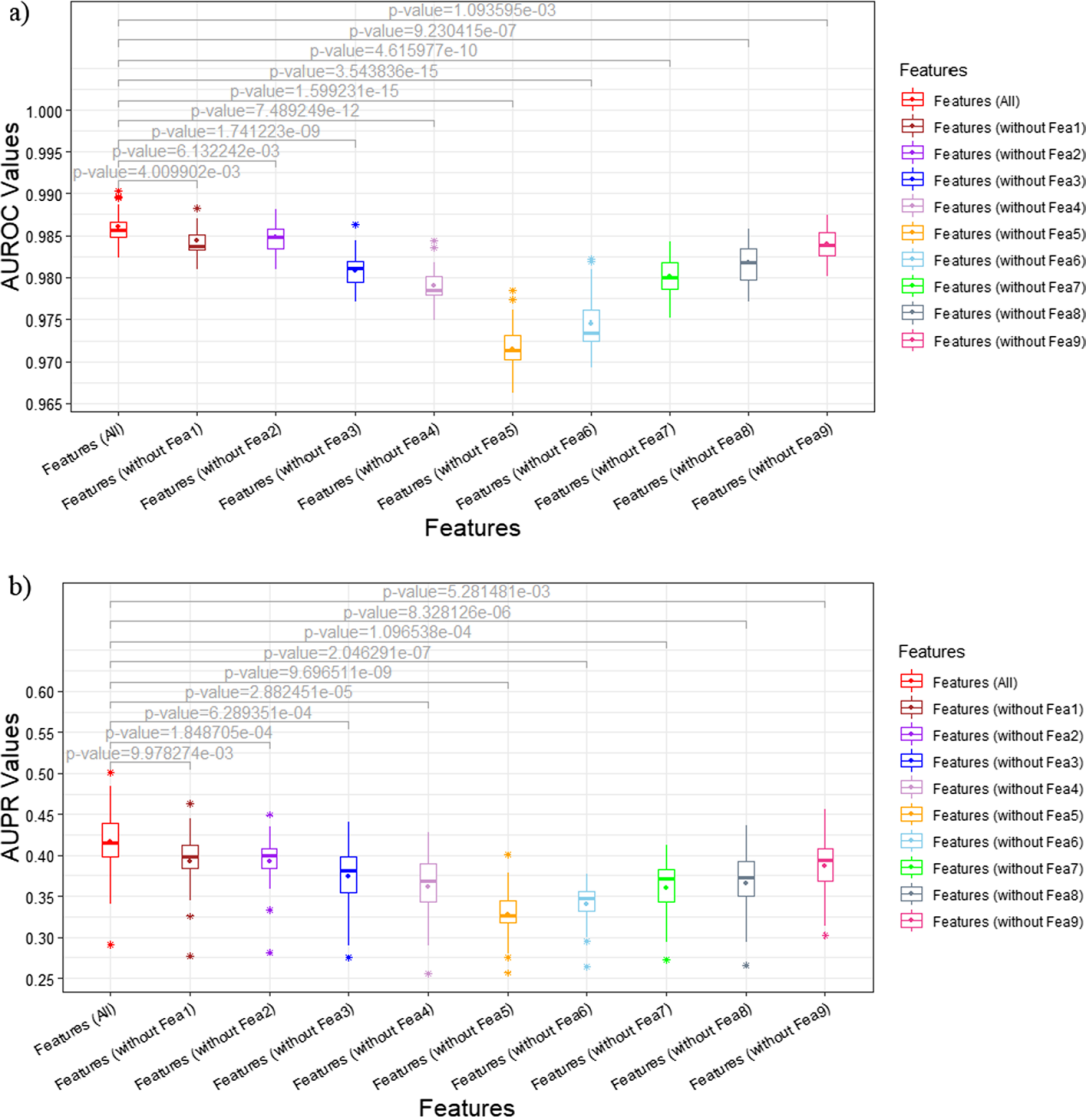
Analysis of features in M2PP. **a** AUROC values via all features and removing each feature with p-values of paired t-test, where the point in the box represent the mean value; **b** AUPR values via all features and removing each feature with p-values of paired t-test, where the point in the box represent the mean value

M2PP acquired mean AUROC of 0.986 and mean AUPR of 0.417 under fivefold CV for 5 times. To detect the influence of features on model’s prediction performance, we removed each feature in turn to run M2PP with the remaining features under the same fold settings. After removing the investigated feature, the more reduced the prediction performance, the more effective the feature was. The AUROC and AUPR values via removing different feature were exhibited by boxplots in Fig. 2, where the mean values were represented by point in the box. It could be observed that the mean AUROC/AUPR values of using all features was better than removing any feature. The paired t-test [33] was performed between AUROC (AUPR) values of using all features and values of removing any feature to check whether the average difference in their performance is significantly different from zero. All p-values were less than 0.05 as shown in Fig. 2, indicating that the performance of using all features is significantly better than removing any feature. This result demonstrated that each feature was indispensable. To further explore the influence of different feature on prediction performance, we defined an indicator named influence coefficient as below:

29 $${\text{Influence coefficient of Fea}}i = mean\left( {{\text{DifferenceAUROC}}_{i} ,{\text{DifferenceAUPR}}_{i} } \right)$$

$${\text{DifferenceAUROC}}_{i} = 1/\left( {1 + e^{{ - sum\left( {AUROC_{all\, features} - AUROC_{all\, features\backslash Feai} } \right)}} } \right)$$

$${\text{DifferenceAUPR}}_{i} = 1/\left( {1 + e^{{ - sum\left( {AUPR_{all\,features} - AUPR_{all\, features\backslash Feai} } \right)}} } \right)$$

where $1 \le {\text{i}} \le 9$; $AUROC_{all features}$ and $AUPR_{all features}$ represented the AUROC and AUPR values of five times fivefold CV by using all features, respectively;$AUROC_{all features\backslash Feai}$ and $AUPR_{all features\backslash Feai}$ represented the AUROC and AUPR values of five times fivefold CV by removing feature $Feai$, respectively. The larger the influence coefficient, the more effective the feature was. The influence coefficient of each feature were shown in Table 2. In the neighborhood similarity information type, Fea3 got the largest influence coefficient, because Fea3 mainly utilized the nearest neighborhoods’ similarity, which was the most valid information in similarity networks. In the path information type, Fea5 and Fea6 obtained advantageous influence coefficients, because paths of length = 2 provided more basic, direct and non-redundant information than length = 3. The drug-inferred information type, Fea4, also acquired decent influence coefficient, indicating that drug indeed play an effective role in predicting target-disease associations because of the drug-target-disease mechanism. Hence, our constructed features were effective, reasonable and indispensable to achieve excellent prediction performance.

### Comparison with existing prediction models

**Fig. 3:**
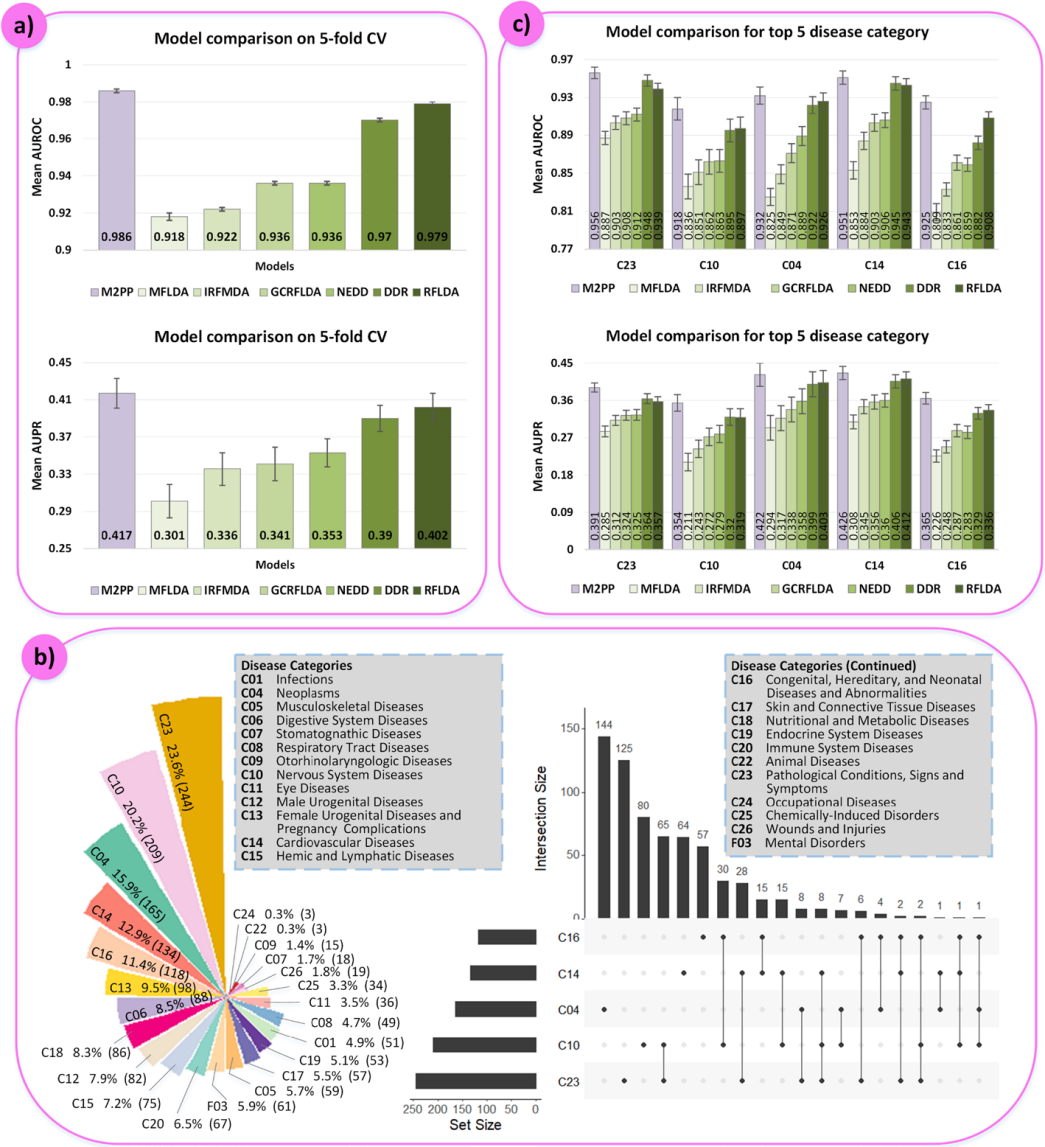
Model comparison. **a** Models’ prediction performance on 5-fold CV for five times, including the mean AUROC/AUPR values with SD marked at the top of each bar; **b** Statistics of disease categories, including the circular bar chart in the left side to exhibit the number and proportion of diseases in each category and the UpSet chart in the right side to exhibit the details of diseases in the top 5 category; **c** Models’ prediction performance for the top 5 category, including the mean AUROC/AUPR values with SD marked at the top of each bar

M2PP was compared with six state-of-the-art models, which were RFLDA [34], DDR [35], NEDD [36], IRFMDA [37], GCRFLDA [38] and MFLDA [39]. The first four methods were based on random forest algorithm, and the last two methods were based on the graph convolutional matrix completion and the matrix factorization, respectively. We performed fivefold CV for five times on each model, exhibiting the mean and SD of AUROC/AUPR values in Fig. 3a). The AUROC values were 0.986 ± 0.001 (M2PP), 0.918 ± 0.002 (MFLDA), 0.922 ± 0.001 (IRFMDA), 0.936 ± 0.001 (GCRFLDA), 0.936 ± 0.001 (NEDD), 0.97 ± 0.001 (DDR) and 0.979 ± 0.001 (RFLDA); the AUPR values were 0.417 ± 0.016 (M2PP), 0.301 ± 0.018 (MFLDA), 0.336 ± 0.017 (IRFMDA), 0.341 ± 0.018 (GCRFLDA), 0.353 ± 0.015 (NEDD), 0.39 ± 0.014 (DDR) and 0.402 ± 0.015 (RFLDA). Whether AUROC or AUPR values, M2PP always achieved the advantageous performance among all methods.

Each disease belonged to at least one category provided by MESH, for example, disease “Lymphoma” belonged to three categories, which were “C04: Neoplasms”, “C15: Hemic and Lymphatic Diseases” and “C20: Immune System Diseases”. In our network, diseases involved 24 categories, where the number and proportion of diseases in each category were shown in the left graph in Fig. 3b). Proportion of the top 5 category “C23: Pathological Conditions, Signs and Symptoms”, “C10: Nervous System Diseases”, “C04: Neoplasms”, “C14: Cardiovascular Diseases” and “C16: Congenital, Hereditary, and Neonatal Diseases and Abnormalities” exceeded 10%, whose UpSet chart was shown in the right side in Fig. 3b) to exhibit the details of diseases in them. For these five categories, we detected models’ prediction performance for their diseases. First, we trained the model with a training sample set which included known target-disease (excluded diseases in the investigated category) associations as the positive samples and the randomly selected unconfirmed target-disease (excluded diseases in the investigated category) pairs as the negative samples, noting that the number of positive and negative samples were the same. Second, the pairs between all targets and each disease in the investigated category were considered as the test set in turn to acquire scores by the model. Then, we could compute the AUROC and AUPR values for each disease in the investigated category, and the average AUROC/AUPR value was considered as the prediction performance of the investigated category. The process was repeated for 5 times to get reliable results. Each model’s mean and SD of AUROC/AUPR values for the five categories were exhibited in Fig. 3c), where M2PP always achieved the best performance. These results indicated the excellent ability of our model.

### Case studies

We predicted new pathogenic proteins for five common diseases: lung cancer, breast cancer, colon cancer, leukemia and lymphoma. For one investigated disease, M2PP was trained with a training sample set, where the known target-disease (excluded the investigated disease) associations was the positive samples and the randomly selected unconfirmed target-disease (excluded the investigated disease) pairs of the same size was the negative samples. Then, M2PP could predict for the pairs between all targets and the investigated disease to acquire prediction scores. We repeated the process for 5 times, so the pair between one target and the investigated disease had five scores, and finally the average score was considered as the prediction score of the pair. We sorted the prediction score of all unconfirmed pairs between targets and the investigated disease, and manually searched the top 10 pairs in public biomedical literature to find the supporting evidence. All top 10 targets were successfully predicted for lung cancer, breast cancer and colon cancer, nine targets for leukemia and seven targets for lymphoma, shown in Table 3. Here, we mainly introduced the top 1 predicted target for each disease. Researchers found that TNF played a key role in inducing resistance to epidermal growth factor receptor inhibition in lung cancer, and suggested that a concomitant inhibition of epidermal growth factor receptor and TNF maybe a potentially new treatment strategy for lung cancer patients [40]. IL2 inhibited the growth of breast cancer cells through improving the proliferation of natural killer cells [41]. Inhibiting or knocking MET down made colon cancer cells sensitive on cetuximab-mediated growth inhibition, implicating that targeting MET was a rational strategy for reversing cetuximab resistance in colon cancer [42]. VEGFA was observed to have additive effect in inflating the risk of leukemia [43]. CHKA possessed oncogenic activity and could be a potential therapeutic target in lymphoma [44]. We also predicted target-disease association scores on the whole network and sorted all unconfirmed pairs’ scores. Seven associations in top 10 has been successfully predicted with public literature as evidences, shown in Table 4. For example, researchers investigated the expression and functions of ALOX5 in breast cancer cells, and demonstrated that inhibiting ALOX5 had therapeutic potential in breast cancer [45]. In addition to these literature evidences, we also found that no matter in Tables 3 or 4, targets and diseases in all successful predictions had co-associated drugs (CDs), which were drugs simultaneously associated with the target and disease. The phenomenon further demonstrated that these high-rank predicted pairs were reasonable from the aspect of both computational data and biomedicine verification. Other drugs which interacted with the predicted target might be potential candidate therapeutic strategies for the investigated disease, needing to be explored in future clinical trials. These results indicated the ability of M2PP to provide conveniences for the future biological researches.

**Table 3:** Successfully predicted pathogenic targets in top 10 for common diseases

Disease name	Rank	Target name	Have CDs	Evidence	Disease name	Rank	Target name	Have CDs	Evidence
Lung cancer	1	TNF	Yes	[40]	Colon cancer	4	ESR1	Yes	[46]
	2	IL1B	Yes	[47]		5	ACE	Yes	[48, 49]
	3	CTNNB1	Yes	[50, 51]		6	CYP2A6	Yes	[52]
	4	ESR1	Yes	[53]		7	CA1	Yes	[54]
	5	MMP9	Yes	[55]		8	PIK3CA	Yes	[56]
	6	MAPK3	Yes	[57]		9	PLAU	Yes	[58]
	7	SOD1	Yes	[59]		10	CYP2E1	Yes	[60]
	8	AKT1	Yes	[61]	Leukemia	1	VEGFA	Yes	[43]
	9	MAPK1	Yes	[62]		2	HIF1A	Yes	[63]
	10	PTGS2	Yes	[64]		4	TGM2	Yes	[65]
Breast cancer	1	IL2	Yes	[41]		5	JUN	Yes	[66]
	2	NR3C1	Yes	[67]		6	TP53	Yes	[68]
	3	PON1	Yes	[69, 70]		7	AKT1	Yes	[71]
	4	JAK2	Yes	[72]		8	GSTP1	Yes	[73, 74]
	5	ICAM1	Yes	[75]		9	CDK4	Yes	[76]
	6	VEGFA	Yes	[77]		10	SMO	Yes	[78]
	7	CCL2	Yes	[79]	Lymphoma	1	CHKA	Yes	[44]
	8	ADRB2	Yes	[80]		3	BCL2	Yes	[81]
	9	PLAU	Yes	[82, 83]		4	GSTP1	Yes	[84, 85]
	10	B2M	Yes	[86]		5	HMOX1	Yes	[87]
Colon cancer	1	MET	Yes	[42]		6	ATP6V1B2	Yes	[88]
	2	NOS3	Yes	[89]		7	TP53	Yes	[90, 91]
	3	ESR2	Yes	[92]		8	VEGFA	Yes	[93]

**Table 4:** Successfully predicted target-disease associations on the whole network in top 10

Target name	Disease name	Have CDs	Rank	Evidence
ALOX5	Breast cancer	Yes	1	[45]
NQO1	Lung cancer	Yes	2	[94]
MMP14	Non-small-cell lung cancer	Yes	3	[95, 96]
BRAF	Breast cancer	Yes	5	[97]
ERBB2	Colon cancer	Yes	6	[98, 99]
MMP14	Stomach cancer	Yes	8	[100]
ERBB2	Hepatocellular Cancer	Yes	10	[101, 102]

## Conclusion

Predicting drug-targeted pathogenic proteins is crucial for understanding disease mechanism and implementing disease treatment. In this study, we presented a novel model M2PP to predict drug-targeted pathogenic proteins. First, we constructed a heterogeneous network, including the target-disease association network, target-drug interaction network, disease-drug association network, disease-disease similarity network, target-target similarity network and drug-drug topological structure similarity network. Then, we developed three types of features on the network, which were based on neighborhood similarity information, drug-inferred information and path information. Finally, we trained a random forest model with these features to score unconfirmed target-disease pairs. In the result section, we first analyzed our constructed features in detail. By removing each feature in turn to check the change of prediction performance, we found that each feature was indispensable. Three types of feature obtained the average influence coefficient of 0.598 (the neighborhood similarity information), 0.671 (the drug-inferred information type) and 0.677 (the path information type), respectively. The path information type acquired the highest value mainly benefited from paths of length = 2, which provided more basic, direct and non-redundant information than paths of length = 3. In addition, the drug-inferred information type also got decent value, indicating that drugs were effective in predicting target-disease associations because of the drug-target-disease mechanism. Then, we compared M2PP with several state-of-the-art models, where M2PP obtained advantageous performance among them. According to the disease category, we extracted sub-networks from the whole target-disease association network for the top 5 category to perform the prediction. Results showed that category of “C23”, “C04” and “C14” achieved better performance. This was because that diseases in “C23”, “C04” and “C14” have more associations with targets than in the other two categories “C10” and “C16”. The average degree of diseases in “C23”, “C04” and “C14” were 6.84 (1670 associations /244 diseases), 12.03 (1985/165) and 7.16 (960/134); while in “C10” and “C16”, the average degree of diseases were 5.06 (1057/209) and 2.95 (348/118). Finally, we predicted new target-disease associations using M2PP, where several high rank associations were successfully confirmed with public literature as evidence. These results demonstrated that M2PP was effective and accurate, which might be convenient for biological researches in the future.

## Data Availability

All data and materials in our manuscript were available on: DrugBank (https://go.drugbank.com/), CTD (http://ctdbase.org/), MESH (https://meshb.nlm.nih.gov/search), DO (https://disease-ontology.org/) and KEGG (https://www.kegg.jp/). The original data and code of M2PP is available at https://github.com/shimingwang1994/M2PP.git

## Abbreviations

PPI: Protein–protein interaction
FDA: Food and drug administration
CV: Cross-validation
AUROC: The areas under the receiver operating characteristic curve
AUPR: The areas under the precision–recall curve
SD: Standard deviation
CDs: Co-associated drugs
CTD: Comparative toxicogenomics database
DO: Disease ontology

## References

[1] Hong K-W, Oh B-S (2010). Overview of personalized medicine in the disease genomic era. BMB Rep.

[2] Giallourakis C, Henson C, Reich M, Xie X, Mootha VK (2005). Disease gene discovery through integrative genomics. Annu Rev Genomics Hum Genet.

[3] Hurle MR, Yang L, Xie Q, Rajpal DK, Sanseau P, Agarwal P (2013). Computational drug repositioning: from data to therapeutics. Clin Pharmacol Ther.

[4] Köhler S, Bauer S, Horn D, Robinson PN (2008). Walking the interactome for prioritization of candidate disease genes. The American Journal of Human Genetics.

[5] Vanunu O, Magger O, Ruppin E, Shlomi T, Sharan R, Wasserman WW: Associating Genes and protein complexes with disease via network propagation. PLOSComput Biol 2010, 6. 10.1371/journal.pcbi.1000641 PMC2797085 20090828

[6] Jia P, Zheng S, Long J, Zheng W, Zhao Z (2011). dmGWAS: dense module searching for genome-wide association studies in protein–protein interaction networks. Bioinformatics.

[7] Wu M, Zeng W, Liu W, Zhang Y, Chen T, Jiang R: Integrating embeddings of multiple gene networks to prioritize complex disease-associated genes. In: 2017 IEEE International Conference on Bioinformatics and Biomedicine (BIBM): 2017. IEEE: 208–215.

[8] Lee I, Blom UM, Wang PI, Shim JE, Marcotte EM (2011). Prioritizing candidate disease genes by network-based boosting of genome-wide association data. Genome Res.

[9] Hou L, Chen M, Zhang CK, Cho J, Zhao H (2014). Guilt by rewiring: gene prioritization through network rewiring in genome wide association studies. Hum Mol Genet.

[10] Luo P, Tian L-P, Ruan J, Wu F-X (2017). Disease gene prediction by integrating ppi networks, clinical rna-seq data and omim data. IEEE/ACM Trans Comput Biol Bioinf.

[11] Wang Q, Yu H, Zhao Z, Jia P (2015). EW_dmGWAS: edge-weighted dense module search for genome-wide association studies and gene expression profiles. Bioinformatics.

[12] Nam Y, Jhee JH, Cho J, Lee J-H, Shin H (2019). Disease gene identification based on generic and disease-specific genome networks. Bioinformatics.

[13] Luo P, Tian L-P, Chen B, Xiao Q, Wu F-X (2020). Ensemble disease gene prediction by clinical sample-based networks. BMC Bioinformatics.

[14] Tomczak K, Czerwińska P, Wiznerowicz M (2015). The Cancer genome atlas (TCGA): an immeasurable source of knowledge. Contemp Oncol.

[15] Han P, Yang P, Zhao P, Shang S, Liu Y, Zhou J, Gao X, Kalnis P: GCN-MF: disease-gene association identification by graph convolutional networks and matrix factorization. In: P*r*oceedings of the 25th ACM SIGKDD international conference on knowledge discovery & data mining*: 2019*. 705–713.

[16] Natarajan N, Dhillon IS (2014). Inductive matrix completion for predicting gene–disease associations. Bioinformatics.

[17] Singh-Blom UM, Natarajan N, Tewari A, Woods JO, Dhillon IS, Marcotte EM: Prediction and validation of gene-disease associations using methods inspired by social network analyses. PloS one 2013, 8(5):e58977. 10.1371/journal.pone.0058977 PMC3641094 23650495

[18] Zeng X, Ding N, Rodríguez-Patón A, Zou Q (2017). Probability-based collaborative filtering model for predicting gene–disease associations. BMC Med Genomics.

[19] Luo P, Li Y, Tian L-P, Wu F-X (2019). Enhancing the prediction of disease–gene associations with multimodal deep learning. Bioinformatics.

[20] Wishart DS, Craig K, Guo AC, Cheng D, Savita S, Dan T, Bijaya G, Murtaza H: DrugBank: a knowledgebase for drugs, drug actions and drug targets. Nucleic Acids Res 2008, 36(suppl_1):D901-D906. 10.1093/nar/gkm958 PMC2238889 18048412

[21] Paul SM, Mytelka DS, Dunwiddie CT, Persinger CC, Munos BH, Lindborg SR, Schacht AL: How to improve R&D productivity: the pharmaceutical industry's grand challenge. Nat Rev Drug Discov 2010:203–214. 10.1038/nrd3078 20168317

[22] Peter DA, Grondin CJ, Kelley LH, Cynthia SR, Daniela S, King BL, Wiegers TC, Mattingly CJ (2015). The comparative toxicogenomics database's 10th year anniversary: update 2015. Nucleic Acids Res.

[23] Lipscomb CE (2000). Medical subject headings (MeSH). Bull Med Libr Assoc.

[24] Fan W, Shang J, Li F, Sun Y, Liu JX: IDSSIM: an lncRNA functional similarity calculation model based on an improved disease semantic similarity method. BMC Bioinform 2020, 21(1). 10.1186/s12859-020-03699-9 PMC7430881 32736513

[25] Kibbe WA, Arze C, Felix V, Mitraka E, Schriml LM: Disease Ontology 2015 update: An expanded and updated database of Human diseases for linking biomedical knowledge through disease data. Nucleic Acids Research 2014, 43(D1). 10.1093/nar/gku1011 PMC4383880 25348409

[26] Wang JZ, Du Z, Payattakool R, Yu PS, Chen C (2007). A new method to measure the semantic similarity of GO terms. Bioinformatics.

[27] Van Laarhoven T, Nabuurs SB, Marchiori E (2011). Gaussian interaction profile kernels for predicting drug–target interaction. Bioinformatics.

[28] Zhao Y, Chen X, Yin J (2019). Adaptive boosting-based computational model for predicting potential miRNA-disease associations. Bioinformatics.

[29] Kanehisa M, Goto S, Hattori M, Aoki-Kinoshita KF, Hirakawa M: From genomics to chemical genomics: new developments in KEGG. Nucleic Acids Research 2006, 34(Database issue):D354–357. 10.1093/nar/gkj102 PMC1347464 16381885

[30] Smith TF, Waterman MS (1981). Identification of common molecular subsequences. J Mol Biol.

[31] Chen X, Yan C, Luo C, Ji W, Zhang Y, Dai Q (2015). Constructing lncRNA functional similarity network based on lncRNA-disease associations and disease semantic similarity. Sci Rep.

[32] Ho TK: Random decision forests. In: Document Analysis and Recognition, 1995, Proceedings of the Third International Conference on: 1995.

[33] Demšar J (2006). Statistical comparisons of classifiers over multiple data sets. J Mach Learn Res.

[34] Yao D, Zhan X, Zhan X, Kwoh CK, Li P, Wang J (2020). A random forest based computational model for predicting novel lncRNA-disease associations. BMC Bioinform.

[35] Olayan RS, Ashoor H, Bajic VB (2018). DDR: efficient computational method to predict drug–target interactions using graph mining and machine learning approaches. Bioinformatics.

[36] Zhou R, Lu Z, Luo H, Xiang J, Zeng M, Li M (2020). NEDD: a network embedding based method for predicting drug-disease associations. BMC Bioinform.

[37] Yao D, Zhan X, Kwoh C-K (2019). An improved random forest-based computational model for predicting novel miRNA-disease associations. BMC Bioinform.

[38] Fan Y, Chen M, Pan X: GCRFLDA: scoring lncRNA-disease associations using graph convolution matrix completion with conditional random field. Briefings in Bioinformatics 2021. 10.1093/bib/bbab361 34486019

[39] Fu G, Wang J, Domeniconi C, Yu G (2018). Matrix factorization-based data fusion for the prediction of lncRNA–disease associations. Bioinformatics.

[40] Gong K, Guo G, Gerber DE, Gao B, Peyton M, Huang C, Minna JD, Hatanpaa KJ, Kernstine K, Cai L (2018). TNF-driven adaptive response mediates resistance to EGFR inhibition in lung cancer. J Clin Investig.

[41] Widowati W, Jasaputra DK, Sumitro SB, Widodo MA, Mozef T, Rizal R, Kusuma HSW, Laksmitawati DR, Murti H, Bachtiar I (2020). Effect of interleukins (IL-2, IL-15, IL-18) on receptors activation and cytotoxic activity of natural killer cells in breast cancer cell. Afr Health Sci.

[42] Song N, Liu S, Zhang J, Liu J, Xu L, Liu Y, Qu X (2014). Cetuximab-induced MET activation acts as a novel resistance mechanism in colon cancer cells. Int J Mol Sci.

[43] Lakkireddy S, Aula S, Kapley A, Swamy A, Digumarti RR, Kutala VK, Jamil K (2016). Association of vascular endothelial growth factor A (VEGFA) and its receptor (VEGFR2) gene polymorphisms with risk of chronic myeloid leukemia and influence on clinical outcome. Mol Diagn Ther.

[44] Xiong J, Bian J, Wang L, Zhou J, Wang Y, Zhao Y, Wu L, Hu J, Li B, Chen S (2015). Dysregulated choline metabolism in T-cell lymphoma: role of choline kinase-α and therapeutic targeting. Blood Cancer J.

[45] Zhou X, Jiang Y, Li Q, Huang Z, Yang H, Wei C: Aberrant ALOX5 Activation correlates with HER2 status and mediates breast cancer biological activities through multiple mechanisms. BioMed research international 2020, 2020. 10.1155/2020/1703531 PMC7673939 33224971

[46] Liu S, Fan W, Gao X, Huang K, Ding C, Ma G, Yan L, Song S (2019). Estrogen receptor alpha regulates the Wnt/β-catenin signaling pathway in colon cancer by targeting the NOD-like receptors. Cell Signal.

[47] Li C, Wang C (2013). Current evidences on IL1B polymorphisms and lung cancer susceptibility: a meta-analysis. Tumor Biol.

[48] Ozawa T, Hashiguchi Y, Yagi T, Fukushima Y, Shimada R, Hayama T, Tsuchiya T, Nozawa K, Iinuma H, Ishihara S (2019). Angiotensin I-converting enzyme inhibitors/angiotensin II receptor blockers may reduce tumor recurrence in left-sided and early colorectal cancers. Int J Colorectal Dis.

[49] Makar GA, Holmes JH, Yang Y-X: Angiotensin-converting enzyme inhibitor therapy and colorectal cancer risk. JNCI 2014, 106(2). 10.1093/jnci/djt374 PMC3952198 24431411

[50] Romero AM, Tafe L: CTNNB1 mutations and co-mutations in non-small cell lung cancer. In: Laboratory investigation: 2020. Nature publishing group 75 VARICK ST, 9TH FLR, NEW YORK, NY 10013–1917 USA: 1805–1806.

[51] Zhou C, Li W, Shao J, Zhao J, Chen C (2020). Analysis of the clinicopathologic characteristics of lung adenocarcinoma with CTNNB1 mutation. Front Genet.

[52] Matsuda Y, Saoo K, Yamakawa K, Yokohira M, Suzuki S, Kuno T, Kamataki T, Imaida K (2007). Overexpression of CYP2A6 in human colorectal tumors. Cancer Sci.

[53] Li J, Ji Z, Luo X, Li Y, Yuan P, Long J, Shen N, Lu Q, Zeng Q, Zhong R: Urinary bisphenol A and its interaction with ESR1 genetic polymorphism associated with non-small cell lung cancer: findings from a case-control study in Chinese population. *Chemosphere* 2020, 254:126835. 10.1016/j.chemosphere.2020.126835 32348927

[54] Bekku S, Mochizuki H, Yamamoto T, Ueno H, Takayama E, Tadakuma T (2000). Expression of carbonic anhydrase I or II and correlation to clinical aspects of colorectal cancer. Hepatogastroenterology.

[55] Cheng X, Yang Y, Fan Z, Yu L, Bai H, Zhou B, Wu X, Xu H, Fang M, Shen A (2015). MKL1 potentiates lung cancer cell migration and invasion by epigenetically activating MMP9 transcription. Oncogene.

[56] Voutsadakis IA: The Landscape of PIK3CA Mutations in Colorectal Cancer. Clinical Colorectal Cancer 2021. 10.1016/j.clcc.2021.02.003 33744168

[57] Blackhall FH, Pintilie M, Michael M, Leighl N, Feld R, Tsao M-S, Shepherd FA (2003). Expression and prognostic significance of kit, protein kinase B, and mitogen-activated protein kinase in patients with small cell lung cancer. Clin Cancer Res.

[58] Belaguli NS, Aftab M, Rigi M, Zhang M, Albo D, Berger DH: GATA6 promotes colon cancer cell invasion by regulating urokinase plasminogen activator gene expression. *Neoplasia* 2010, 12(11):856-IN851. 10.1593/neo.10224 PMC2978909 21076612

[59] Somwar R, Erdjument-Bromage H, Larsson E, Shum D, Lockwood WW, Yang G, Sander C, Ouerfelli O, Tempst PJ, Djaballah H (2011). Superoxide dismutase 1 (SOD1) is a target for a small molecule identified in a screen for inhibitors of the growth of lung adenocarcinoma cell lines. Proc Natl Acad Sci.

[60] Morita M, Le Marchand L, Kono S, Yin G, Toyomura K, Nagano J, Mizoue T, Mibu R, Tanaka M, Kakeji Y (2009). Genetic polymorphisms of CYP2E1 and risk of colorectal cancer: the Fukuoka Colorectal Cancer Study. Cancer Epidemiol Prevent Biomark.

[61] Wu H, Liu HY, Liu WJ, Shi YL, Bao D (2019). miR-377-5p inhibits lung cancer cell proliferation, invasion, and cell cycle progression by targeting AKT1 signaling. J Cell Biochem.

[62] Zhang Z-y, Gao X-h, Ma M-y, Zhao C-l, Zhang Y-l, Guo S-s (2020). CircRNA_101237 promotes NSCLC progression via the miRNA-490-3p/MAPK1 axis. Sci Rep.

[63] Kontos CK, Papageorgiou SG, Diamantopoulos MA, Scorilas A, Bazani E, Vasilatou D, Gkontopoulos K, Glezou E, Stavroulaki G, Dimitriadis G (2017). mRNA overexpression of the hypoxia inducible factor 1 alpha subunit gene (HIF1A): An independent predictor of poor overall survival in chronic lymphocytic leukemia. Leuk Res.

[64] Rausch SM, Gonzalez BD, Clark MM, Patten C, Felten S, Liu H, Li Y, Sloan J, Yang P (2012). SNPs in PTGS2 and LTA predict pain and quality of life in long term lung cancer survivors. Lung Cancer.

[65] Mohammadzadeh Z, Omidkhoda A, Chahardouli B, Hoseinzadeh G, Moghaddam KA, Mousavi SA, Rostami S (2021). The impact of ICAM-1, CCL2 and TGM2 gene polymorphisms on differentiation syndrome in acute promyelocytic leukemia. BMC Cancer.

[66] Zhou C, Martinez E, Di Marcantonio D, Solanki-Patel N, Aghayev T, Peri S, Ferraro F, Skorski T, Scholl C, Fröhling S (2017). JUN is a key transcriptional regulator of the unfolded protein response in acute myeloid leukemia. Leukemia.

[67] Mamoor S: Differential expression of nuclear receptor subfamily 3 group C member 1 in cancers of the breast. 2021.

[68] Prochazka KT, Pregartner G, Rücker FG, Heitzer E, Pabst G, Wölfler A, Zebisch A, Berghold A, Döhner K, Sill H (2019). Clinical implications of subclonal TP53 mutations in acute myeloid leukemia. Haematologica.

[69] Wen Y, Huang Z, Zhang X, Gao B, He Y (2015). Correlation between PON1 gene polymorphisms and breast cancer risk: a Meta-analysis. Int J Clin Exp Med.

[70] Bobin-Dubigeon C, Jaffré I, Joalland M-P, Classe J-M, Campone M, Hervé M, Bard J-M (2012). Paraoxonase 1 (PON1) as a marker of short term death in breast cancer recurrence. Clin Biochem.

[71] Küçükcankurt F, Erbilgin Y, Fırtına S, Ng ÖH, Karakaş Z, Celkan T, Ünüvar A, Özbek U, Sayitoğlu M (2020). PTEN and AKT1 variations in childhood T-Cell acute lymphoblastic leukemia. Turkish J Hematol.

[72] Kim JW, Gautam J, Kim JE, Kim J, Kang KW (2019). Inhibition of tumor growth and angiogenesis of tamoxifen-resistant breast cancer cells by ruxolitinib, a selective JAK2 inhibitor. Oncol Lett.

[73] Elhoseiny S, El-Wakil M, Fawzy M, Rahman AA (2013). GSTP1 (Ile105Val) gene polymorphism: risk and treatment response in chronic myeloid leukemia. J Cancer Ther.

[74] Kagita Sailaja D, Rao DN, Rao DR, Vishnupriya S (2010). Association of the GSTP1 gene (Ile105Val) polymorphism with chronic myeloid leukemia. Asian Pac J Cancer Prev.

[75] Rosette C, Roth RB, Oeth P, Braun A, Kammerer S, Ekblom J, Denissenko MF (2005). Role of ICAM1 in invasion of human breast cancer cells. Carcinogenesis.

[76] Sawai CM, Freund J, Oh P, Ndiaye-Lobry D, Bretz JC, Strikoudis A, Genesca L, Trimarchi T, Kelliher MA, Clark M (2012). Therapeutic targeting of the cyclin D3: CDK4/6 complex in T cell leukemia. Cancer Cell.

[77] Zou G, Zhang X, Wang L, Li X, Xie T, Zhao J, Yan J, Wang L, Ye H, Jiao S (2020). Herb-sourced emodin inhibits angiogenesis of breast cancer by targeting VEGFA transcription. Theranostics.

[78] Shah NP, Cortes JE, Martinelli G, Smith BD, Clarke E, Copland M, Strauss L, Talpaz M (2014). Dasatinib plus smoothened (SMO) inhibitor BMS-833923 in chronic myeloid leukemia (CML) with resistance or suboptimal response to a prior tyrosine kinase inhibitor (TKI): phase I study CA180323.

[79] Bonapace L, Coissieux M-M, Wyckoff J, Mertz KD, Varga Z, Junt T, Bentires-Alj M (2014). Cessation of CCL2 inhibition accelerates breast cancer metastasis by promoting angiogenesis. Nature.

[80] Feigelson HS, Teras LR, Diver WR, Tang W, Patel AV, Stevens VL, Calle EE, Thun MJ, Bouzyk M (2008). Genetic variation in candidate obesity genes ADRB2, ADRB3, GHRL, HSD11B1, IRS1, IRS2, and SHC1 and risk for breast cancer in the Cancer Prevention Study II. Breast Cancer Res.

[81] Correia C, Schneider PA, Dai H, Dogan A, Maurer MJ, Church AK, Novak AJ, Feldman AL, Wu X, Ding H (2015). BCL2 mutations are associated with increased risk of transformation and shortened survival in follicular lymphoma. Blood J Am Soc Hematol.

[82] Jin H, Choi H, Kim ES, Lee HH, Cho H, Moon A (2021). Natural killer cells inhibit breast cancer cell invasion through downregulation of urokinase-type plasminogen activator. Oncol Rep.

[83] Belfiore L, Saunders DN, Ranson M, Vine KL (2020). N-alkylisatin-loaded liposomes target the urokinase plasminogen activator system in breast cancer. Pharmaceutics.

[84] Ibrahim NY, Sami RM, Nasr AS (2012). GSTP1 and CYP1A1 gene polymorphisms and non-hodgkin lymphoma. Lab Med.

[85] Nakamichi I, Tomita Y, Zhang B, Sugiyama H, Kanakura Y, Fukuhara S, Hino M, Kanamaru A, Ogawa H, Aozasa K (2007). Correlation between promoter hypermethylation of GSTP1 and response to chemotherapy in diffuse large B cell lymphoma. Ann Hematol.

[86] Weiss M, Michael J, Pesce A, DiPersio L (1981). Heterogeneity of beta 2-microglobulin in human breast carcinoma. Lab Invest J Tech Methods Pathol.

[87] Nakashima M, Watanabe M, Nakano K, Uchimaru K, Horie R: Differentiation of Hodgkin lymphoma cells by reactive oxygen species and regulation by heme oxygenase‐1 through HIF‐1α. Cancer Science 2021. 10.1111/cas.14890 PMC8177765 33738869

[88] Wang F, Gatica D, Ying ZX, Peterson LF, Kim P, Bernard D, Saiya-Cork K, Wang S, Kaminski MS, Chang AE (2019). Follicular lymphoma–associated mutations in vacuolar ATPase ATP6V1B2 activate autophagic flux and mTOR. J Clin Investig.

[89] Jeong S, Kim BG, Kim DY, Kim BR, Kim JL, Park SH, Na YJ, Jo MJ, Yun HK, Jeong YA (2019). Cannabidiol overcomes oxaliplatin resistance by enhancing NOS3-and SOD2-induced autophagy in human colorectal cancer cells. Cancers.

[90] Eskelund CW, Dahl C, Hansen JW, Westman M, Kolstad A, Pedersen LB, Montano-Almendras CP, Husby S, Freiburghaus C, Ek S (2017). TP53 mutations identify younger mantle cell lymphoma patients who do not benefit from intensive chemoimmunotherapy. Blood J Am Soc Hematol.

[91] Zenz T, Kreuz M, Fuge M, Klapper W, Horn H, Staiger AM, Winter D, Helfrich H, Huellein J, Hansmann ML (2017). TP53 mutation and survival in aggressive B cell lymphoma. Int J Cancer.

[92] Sainz J, Rudolph A, Hein R, Hoffmeister M, Buch S, Von Schönfels W, Hampe J, Schafmayer C, Völzke H, Frank B (2011). Association of genetic polymorphisms in ESR2, HSD17B1, ABCB1, and SHBG genes with colorectal cancer risk. Endocrine Related Cancer.

[93] Mashhadi MA, Arbabi N, Sargazi S, Kazemi-Lomedasht F, Jahantigh D, Miri-Moghaddam E: Association of VEGFA gene polymorphisms with susceptibility to non-Hodgkin's lymphoma: Evidences from population-based and in silico studies. Gene Rep 2020, 20:100696.

[94] Kiyohara C, Yoshimasu K, Takayama K, Nakanishi Y (2005). NQO1, MPO, and the risk of lung cancer: a HuGE review. Genet Med.

[95] Wang Y-Z, Wu K-P, Wu A-B, Yang Z-C, Li J-M (2014). Mo Y-l, Xu M, Wu B, Yang Z-x: MMP-14 overexpression correlates with poor prognosis in non-small cell lung cancer. Tumor Biol.

[96] Zhou H, Wu A, Fu W, Lv Z, Zhang Z (2014). Significance of semaphorin-3A and MMP-14 protein expression in non-small cell lung cancer. Oncol Lett.

[97] Jung YY, Jung WH, Koo JS: BRAF mutation in breast cancer by BRAF V600E mutation-specific antibody. 2016.

[98] Kloth M, Ruesseler V, Engel C, Koenig K, Peifer M, Mariotti E, Kuenstlinger H, Florin A, Rommerscheidt-Fuss U, Koitzsch U (2016). Activating ERBB2/HER2 mutations indicate susceptibility to pan-HER inhibitors in Lynch and Lynch-like colorectal cancer. Gut.

[99] Maurer CA, Friess H, Kretschmann B, Zimmermann A, Stauffer A, Baer HU, Korc M, Buchler MW (1998). Increased expression of erbB3 in colorectal cancer is associated with concomitant increase in the level of erbB2. Hum Pathol.

[100] Zhuoyu G, Siyuan L, Xiao Z, Zhou T, Jun L (2015). Expression and role of MMP-14 protein in invasion and metastasis of stomach carcinoma. Chongqing Med.

[101] Wong CI, Yap HL, Lim SG, Guo JY, Goh BC, Lee SC (2008). Lack of somatic ErbB2 tyrosine kinase domain mutations in hepatocellular carcinoma. Hepatol Res.

[102] Bekaii-Saab T, Williams N, Plass C, Calero MV, Eng C (2006). A novel mutation in the tyrosine kinase domain of ERBB2 in hepatocellular carcinoma. BMC Cancer.

